# α-Hederin Saponin Augments the Chemopreventive Effect of Cisplatin against Ehrlich Tumors and Bioinformatic Approach Identifying the Role of SDF1/CXCR4/p-AKT-1/NFκB Signaling

**DOI:** 10.3390/ph16030405

**Published:** 2023-03-07

**Authors:** Samah M. Elaidy, Mohamed K. El-Kherbetawy, Sally Y. Abed, Abdullah Alattar, Reem Alshaman, Mohamed Ahmed Eladl, Eman Saad Alamri, Aisha Nawaf Al balawi, AbdelNaser Zaid, Amany Y. Elkazzaz, Sozan M. Abdelkhalig, Ziad E. Hamed, Sawsan A. Zaitone

**Affiliations:** 1 Clinical Pharmacology Department, Faculty of Medicine, Suez Canal University, Ismailia 41522, Egypt; 2 Department of Pathology, Faculty of Medicine, Suez Canal University, Ismailia 41522, Egypt; 3 Department of Respiratory Care, College of Applied Medical Science in Jubail, Imam Abdulrahman Bin Faisal University, Jubail 35816, Saudi Arabia; 4 Department of Pharmacology and Toxicology, Faculty of Pharmacy, University of Tabuk, Tabuk 71491, Saudi Arabia; 5 Department of Basic Medical Sciences, College of Medicine, University of Sharjah, Sharjah 27272, United Arab Emirates; 6 Nutrition and Food Science Department, University of Tabuk, Tabuk 71491, Saudi Arabia; 7 Biology Department, University College of Haqel, University of Tabuk, Tabuk 71491, Saudi Arabia; 8 Department of Surgery, Faculty of Medicine, Jazan University, Jazan 45142, Saudi Arabia; 9 Department of General Surgery, Faculty of Medicine, Assiut University, Assiut 71515, Egypt; 10 Department of Medical Biochemistry and Molecular Biology, Faculty of Medicine, Port-Said University, Port-Said 42526, Egypt; 11 Department of Medical Biochemistry and Molecular Biology, Faculty of Medicine, Suez Canal University, Ismailia 41522, Egypt; 12 Department of Basic Medical Sciences, College of Medicine, AlMaarefa University, Riyadh 13713, Saudi Arabia; 13 Medicine and Surgery Program, Faculty of Medicine, Mansoura University, Mansoura 35511, Egypt

**Keywords:** α-hederin, cisplatin, Ehrlich solid tumor, mouse, SDF1/CXCR4/p-AKT/NFκB signaling

## Abstract

Stromal cell-derived factor-1 (SDF1) and its C-X-C chemokine receptor type 4 receptor (CXCR4) are significant mediators for cancer cells’ proliferation, and we studied their expression in Ehrlich solid tumors (ESTs) grown in mice. α-Hederin is a pentacyclic triterpenoid saponin found in Hedera or Nigella species with biological activity that involves suppression of growth of breast cancer cell lines. The aim of this study was to explore the chemopreventive activity of α-hederin with/without cisplatin; this was achieved by measuring the reduction in tumor masses and the downregulation in SDF1/CXCR4/pAKT signaling proteins and nuclear factor kappa B (NFκB). Ehrlich carcinoma cells were injected in four groups of Swiss albino female mice (Group1: EST control group, Group2: EST + α-hederin group, Group3: EST + cisplatin group, and Group4: EST+α-hederin/cisplatin treated group). Tumors were dissected and weighed, one EST was processed for histopathological staining with hematoxylin and eosin (HE), and the second MC was frozen and processed for estimation of signaling proteins. Computational analysis for these target proteins interactions showed direct-ordered interactions. The dissected solid tumors revealed decreases in tumor masses (~21%) and diminished viable tumor regions with significant necrotic surrounds, particularly with the combination regimens. Immunohistochemistry showed reductions (~50%) in intratumoral NFκβ in the mouse group that received the combination therapy. The combination treatment lowered the SDF1/CXCR4/*p*-AKT proteins in ESTs compared to the control. In conclusion, α-hederin augmented the chemotherapeutic potential of cisplatin against ESTs; this effect was at least partly mediated through suppressing the chemokine SDF1/CXCR4/*p*-AKT/NFκB signaling. Further studies are recommended to verify the chemotherapeutic potential of α-hederin in other breast cancer models.

## 1. Introduction

In women, breast cancer is the most diagnosed life-threatening cancer and the top cause of cancer death [1,2]. Many drugs were established for treating breast cancer but, drug resistance and adverse effects often take place [3]. Ehrlich solid tumors (ESTs) is a highly popular experimental tumor used to test various herbal anticancer medicines [4,5,6]. Ehrlich ascites carcinoma is a hyperdiploid undifferentiated tumor that has fast proliferation, no regression, a high transplantable capacity, and malignant characterization [7]. 

Chemokines have been implicated in the development of the tolerated tumor microenvironment [8]. Stromal-cell-derived factor-1 (SDF1) is also recognized as C-X-C motif chemokine ligand 12, CXCL12, which is a key regulatory chemokine found in both healthy and malignant cells [9]. SDF1 is a high-affinity ligand for the G-protein-coupled receptor C-X-C chemokine receptor type-4 (CXCR4). The chemokine receptor SDF1/CXCR4 promote inflammation, growth, and spread of various cancers, including cervical and breast malignancies [10]. Furthermore, SDF1/CXCR4 communication seemed to connect tumors and their stromal cells [11], as well as activate many transcription factor machineries, including nuclear factor-kappa B (NFκB) [12] and protein kinase B (AKT) [13,14].

NFκB transcription factors were shown to have an important role in a variety of malignancies. NFκB through its transcription apparatus can influence a variety of inflammatory mediators, apoptosis, stress responses, and cellular differentiation [15,16]. Moreover, NFκB pathway activation has vital roles in tumor development and proliferation and angiogenesis [17,18]. The inhibition of NFκB signaling paths is exploited as a particular target in experimental treatment to decrease tumor growth, invasion, and metastasis [19,20]. The AKT protein is an interacting prognostic oncogene that is activated by inflammation and damaged DNA. It is overexpressed in a variety of malignancies, promoting cancer cell proliferation and invasiveness, which reveals the preventative and therapeutic potential of AKT inhibition in cancer [21,22,23]. 

Natural plant foods occupy an important position in cancer therapy. Various triterpenoid saponins provide a potential chemopreventive effect against breast cancer [24]. α-hederin {(3β,4α)-3-[[2-O-(6-Deoxy-α-L-mannopyranosyl)-α-L-arabinopyranosyl]oxy]-23-hydroxyolean-12-en-28-oic acid} is a pentacyclic triterpenoid saponin found in Hedera or Nigella species and was reported to show multiple biological activities. Currently, the number of investigations into its biological activity is increasing due to its promising anticancer potential [25]. α-hederin was reported to display cytotoxic potential against many cancer cell lines [24] and experimental malignancies [24,26]. Importantly, the studies using α-hederin in breast cancer animal models are limited, and the mechanism of its chemotherapeutic action have not been fully understood. The anti-inflammatory activity of ivy leaves (Hedera species) dry extract is attributed to the extract’s regulatory influence on the nuclear factor-κB (NFκB) pathway by switching of the inhibitor of NFκB (Iβ) kinase-(IKK) [18,26]. Furthermore, reports on the chemotherapeutic potential of α-hederin are few; these reports focused on biological activity rather than the mechanisms [27].

Cisplatin was reported to possess anti-tumor potential against EST [28]. Cisplatin is a chemical isomer of the molecule PtCI2(NH3)2; Cis-diamminedichloroplatinum (II) [29]. It has a strong anti-neoplastic impact [30]. Its mode of action is based on DNA damage [31]. Although cisplatin is a key antineoplastic medication, it has dose-limiting nephrotoxicity [32].

Throughout the published evidence, the SDF1/CXCR4 expressional levels in EST tissues need to be more enumerated. Moreover, the manipulation of SDF1/CXCR4 by α-hederin was not studied before. The aim of the current study was to investigate the chemotherapeutic potential of the α-hederin saponin and its effect combined with cisplatin in EST grown in mice focusing on the SDF1/CXCR4/pAKT-1/NFκB signaling pathway as a molecular target. Moreover, the interactions between the targeted proteins were analyzed computationally using the STRING database. 

## 2. Results

### 2.1. Effect of α-Hederin and Cisplatin in Reducing the Solid Tumor Masses 

**Figure 1:**
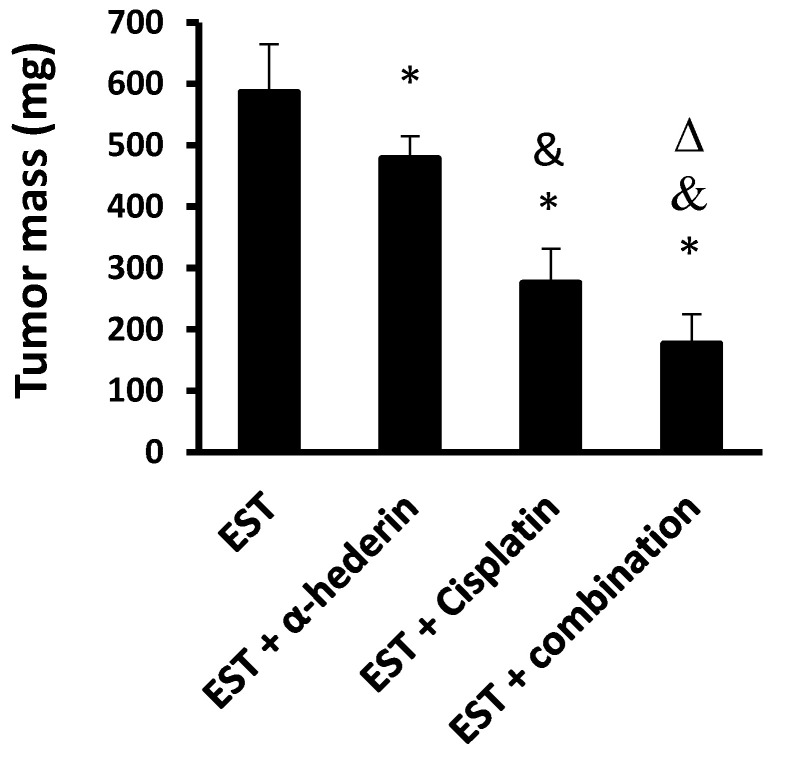
**Effect of α-hederin and cisplatin on solid tumor masses.** EST: Ehrlich solid tumors. Data are mean ± SD, *: versus EST control group, &: versus EST + α-hederin group, Δ: versus EST+ cisplatin group at *p* < 0.05.

The EST control group displayed well-defined solid tumors (0.58 ± 0.78 g in mass). Mice treated with α-hederin, cisplatin, or combination showed quantifiable decreases in tumor masses compared to the EST control mice. In comparison to each treatment alone, the combination cisplatin and α-hederin regimen displayed superior reductions in tumor masses compared to the monotherapies (*p*  <  0.05), Figure 1.

### 2.2. Effect of α-Hederin and Cisplatin on the Level of Inflammatory Factors in ESTs

**Figure 2:**
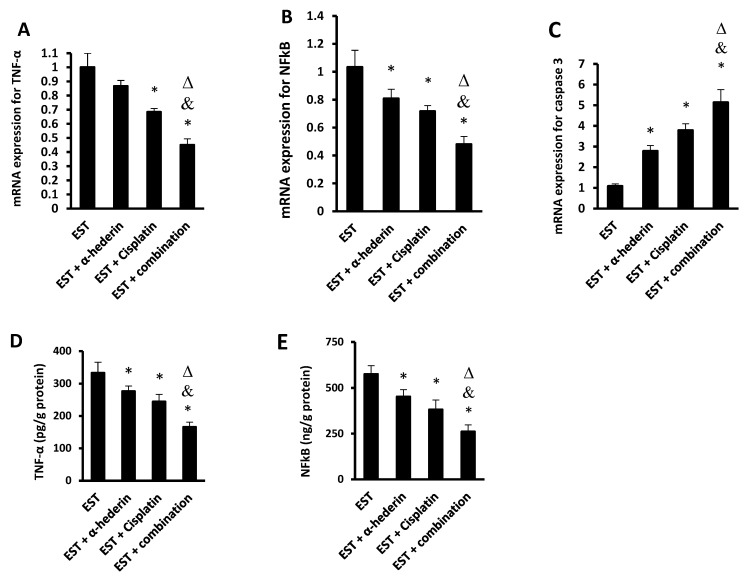
**RT-PCR and protein level for tumor markers.** The mRNA expression folds for (**A**) TNF-α, (**B**) NFκB, and (**C**) caspase 3 and protein level of (**D**) TNF-α, (**E**) NFκB. *: versus EST control, &: versus EST + α-hederin group, Δ: versus EST + cisplatin group. Data are mean ± SD and statistical analyzed at *p* < 0.05.

The mRNA expression of TNF-α in the frozen tumors was significantly reduced in mice treated with cisplatin or the α-hederin/cisplatin combination. The mRNA expression of NFκB was significantly reduced in mice treated with α-hederin, cisplatin, or their combination. The mRNA expression of caspase 3 was significantly increased in mice treated with α-hederin, cisplatin, or their combination. Importantly the expression of these proteins in the combination group was different from their expression in the monotherapy groups (Figure 2A–C). Similarly, the protein level of TNF-α and NFκB in the tumor homogenates was significantly reduced by α-hederin or cisplatin compared to the EST control group. The combination group showed lower cytokine levels compared to the monotherapies, as shown in Figure 2D,E. 

### 2.3. Effect of α-Hederin and Cisplatin on Tumoral Level of SDF1/CXCR4/p-AKT Proteins

**Figure 3:**
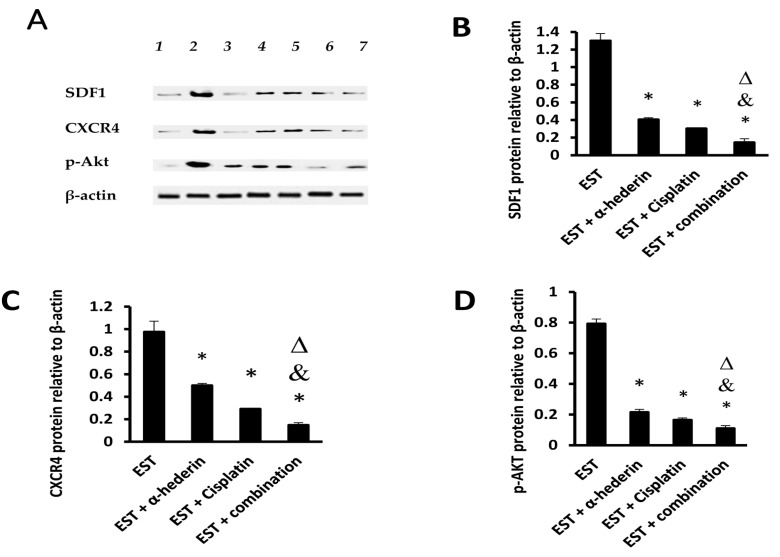
**Western blotting for the target proteins.** Panel (**A**): gels for the (1,3) EST + combination group; (2) EST control group, (4,5) EST + α-hederin group, (6,7) EST + cisplatin group. Panel (**B**): Column chart for SDF1 Panel (**C**): column chart for CXCR4 and Panel (**D**): column chart for p-AKT. *: versus EST control, &: versus EST + α-hederin group, Δ: versus EST + cisplatin group. Data are mean ± SD and statistically analyzed at *p* < 0.05.

In the EST control group, the protein levels of SDF1/CXCR4/*p*-AKT are shown in Figure 3A. Monotherapy with α-hederin or cisplatin significantly reduced the levels of these proteins in comparison to the EST control group. The combination group showed detrimental effect on tumoral SDF1/CXCR4/*p*-AKT proteins in comparison to monotherapy with cisplatin, as shown in Figure 3B–D.

### 2.4. Tumor Characteristics in Sections Stained with Hematoxylin and Eosin

**Figure 4:**
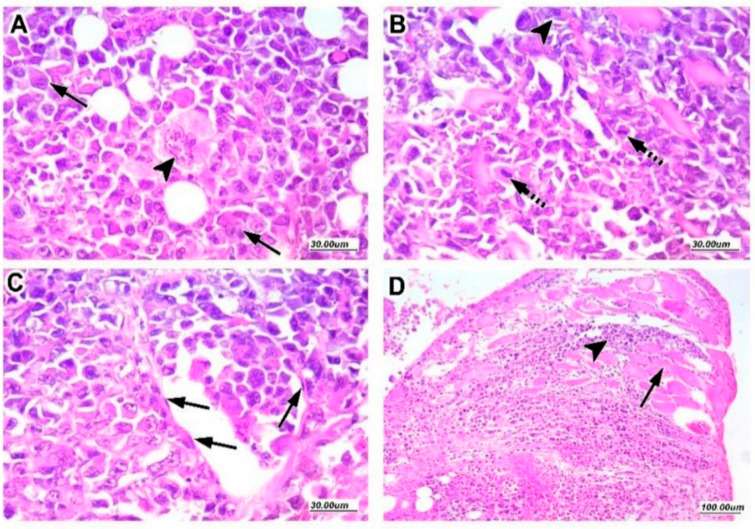
**The pathological characteristics of Ehrlich solid tumors stainied with hematoxylin and eosin.** Images show different tumor characteristics; panel (**A**) tumor cells showing marked nuclear and cellular pleomorphism and hyperchromasia with enlarged nuclei and prominent nucleoli (arrow), with scattered multinucleated tumor giant cells (arrowhead). Panel (**B**) shows multiple mitotic figures (dashed arrow), indicating high mitotic activity with abnormal mitotic figures (arrowhead), a feature of malignant tumor. Panel (**C**) shows lymphovascular invasion by tumor cells; the vessel contour is indicated by arrows pointing out endothelial cell lining of vessel wall. Panel (**D**) is a low-magnification image of tumor cells (arrowhead) infiltrating the surrounding skeletal muscle fibers (arrow).

Tumor histopathology is shown in Figure 4. Panel A–D demonstrated a variety of unusual multiple mitotic tumor features, including significant nuclear pleomorphism with larger nuclei and scattered multinucleated tumor giant cells. Furthermore, malignant cells infiltrated dispersed lymphovascular and skeletal muscle fibers. 

### 2.5. α-Hederin Regressed the Viable Tumor Area in Combination with Cisplatin

**Figure 5:**
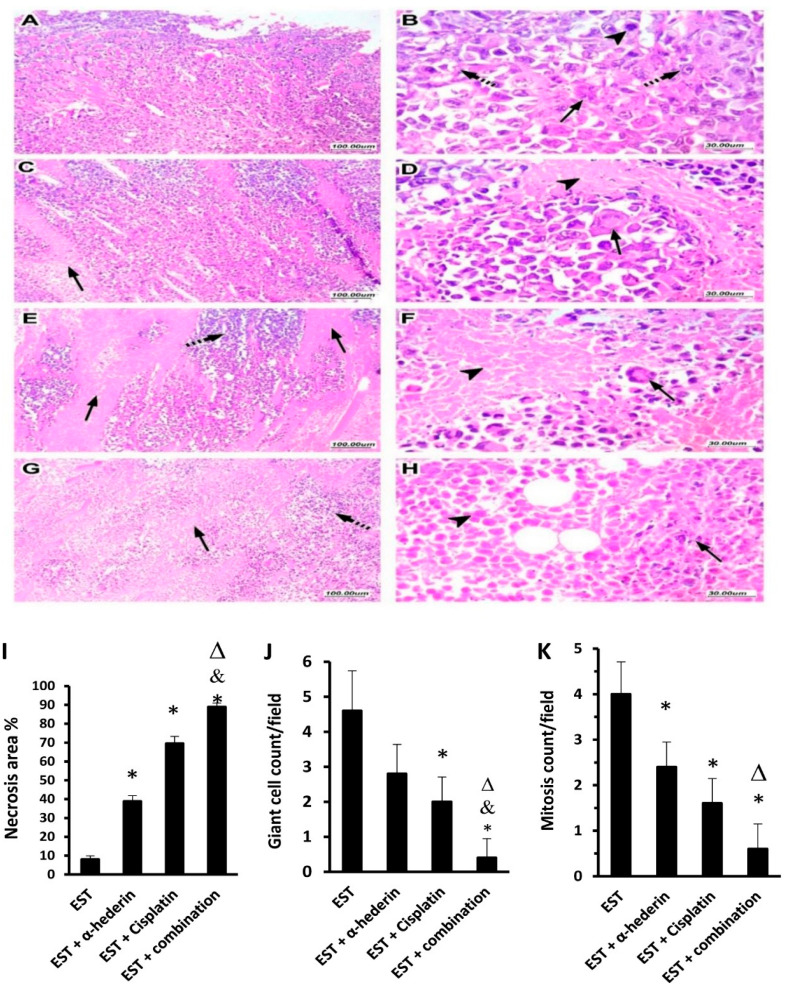
Hematoxylin and eosin staining for solid tumors from the tumor control group and cisplatin groups. Panel (**A**,**B**) show low- and high-magnification images of the control group with viable tumor cells with infiltration of surrounding tissues. Panel (**C**,**D**) show low- and high-magnification images of cisplatin group tumor showing tumor necrosis appearing as granular eosinophilic structureless areas (arrow, panel (**C**)), with central viable tumor cells (arrow, panel (**D**)). Panel (**E**,**F**) low- and high-magnification images of EST + α-hederin group showing areas tumor necrosis (arrow, panel (**E**)), with residual viable tumor cells (dashed arrow, panel (**F**)), with scattered giant cells (arrow, panel (**F**)) and necrotic cells (arrowhead, panel (**F**)). Panel (**G**,**H**) low- and high-magnification images of EST + combination group showing wide areas of necrosis (arrow, panel (**G**)) and scattered very few viable cells (arrow, panel (**H**)). Comparison of the pathological findings in solid tumor sections are shown in (**I**) necrosis area %, (**J**) giant cell count, and (**K**) mitosis count. The pathological findings were determined for six random fields/tumor section and averaged. EST: Ehrlich solid tumors, Data are mean ± SD, *: versus EST control group, &: versus EST + α-hederin group, Δ: versus EST + cisplatin group at *p* < 0.05. Data are mean ± SD and were compared at *p* < 0.05.

Following cisplatin injection, core viable tumor tissue was surrounded by necrotic granular eosinophilic structureless regions, as seen in Figure 5A–D. Treatment with α-hederin, alone or in combination with cisplatin, reduced tumor cell viability significantly, with increased regions of necrosis embedded by ghosts of necrotic tumor cells, as shown in Figure 5, Panel E–H. Further, comparing the experimental groups indicated that EST + α-hederin group showed a 4.85-fold increase, EST + cisplatin group showed an 8.6-fold increase, and the EST + combination group showed an 11.1-fold increase in necrosis area %. (Figure 5I). The giant cell count and mitosis count in the EST + combination group were reduced significantly in comparison to the EST + α-hederin group and the EST + cisplatin (Figure 5J,K). 

### 2.6. Effect of α-Hederin and Cisplatin on Immunostaining for NFκB in ESTs 

**Figure 6:**
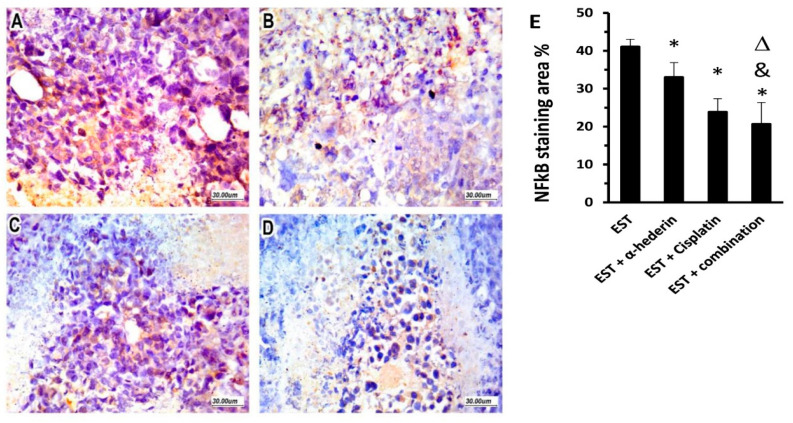
**Immunostaining for NFκB in the study groups**. (**A**) Photomicrograph from the EST control group shows marked expression of NFκB by tumor cells. (**B**) The cisplatin group showed lower expression of NFκB by tumor cells. (**C**) This image shows the EST + α-hederin group with a moderate expression of NFκB by tumor cells with areas of necrosis. (**D**) The combination group, showing lower expression. (**E**) column chart for the staining area %. EST: Ehrlich solid tumors. *: versus EST control group, &: versus EST + α-hederin group, Δ: versus EST + cisplatin group. Data are mean ± SD and were compared at *p* < 0.05.

Untreated tumor tissues showed a marked expression of NFκB, which was mainly allocated in the viable tumor cells, as shown in Figure 6A. Along with reduced viable tumor cells after cisplatin and/or α-hederin treatment, NFκB showed less expressional allocation throughout the residual EST tumor and its necrotic surrounds, as shown in Figure 6B,C. Importantly, the EST + combination group showed the minimal immunostaining for NFκB (Figure 6D). Statistical comparison between the study groups indicated that immunostaining % in the EST + α-hederin group and the EST + cisplatin group (33.02 ± 3.89 & 23.89 ± 3.47) was reduced significantly compared to the EST control group (41.1 ± 1.96, Figure 6E). The EST + combination group showed significantly reduced immunostaining area for NFκB (20.66 ± 5.7) compared to the EST control group, as well as the mice groups that received α-hederin or cisplatin (Figure 6E).

### 2.7. Targeted Protein–Protein Interactions and Analysis of Pathway Enrichment

**Figure 7:**
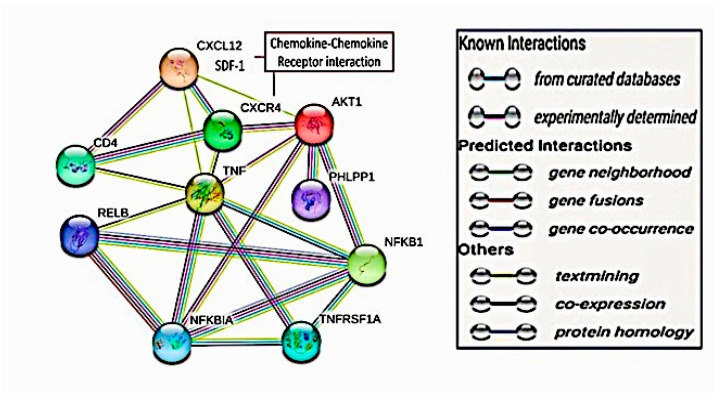
The computational interaction analysis of the target translational proteins related to the chemokine–chemokine receptor interactions. The STRING database was used. The confidence score was 0.700. Network edges showed the evidence interaction types. AKT, protein kinase B; CD4, T-cell surface glycoprotein CD4; CXCR4, C-X-C chemokine receptor type 4 receptor; PHLPP1, PH domain leucine-rich repeat-containing protein phosphatase 1; NFκB; nuclear factor kappa B; NFKBIA, NF-kappa-B inhibitor alpha; RELB, Relb proto-oncogene, NFκB subunit; SDF1, stromal cell-derived factor-1; TNF-α, tumor necrosis factor α; TNFRF1A, tumor necrosis factor receptor superfamily member 1A.

**Figure 8:**
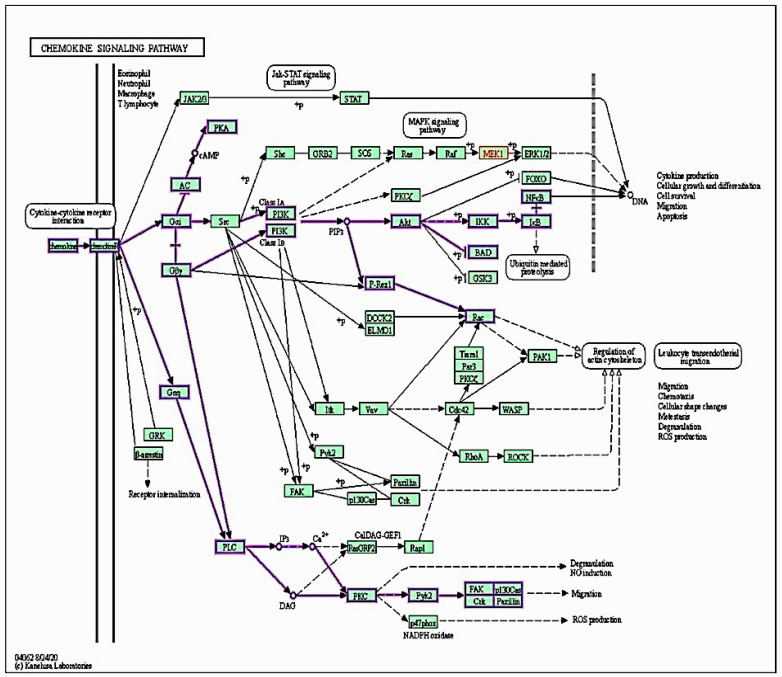
Chemokine signaling pathway. A graph generated by KEGG bioinformatic database.

The targeted proteins showed highly evidenced interactions. SDF1 (CXCL12) showed direct interaction with its receptor CXCR4 with no direct significant interactions to other proteins except for other published evidence for AKT-1 and TNF-α. CXCR4 showed predictive co-expressional interactions with AKT-1 and TNF-α but no predictive interactions with NFκB. These co-expressional relations were also detected experimentally. The AKT-1 exhibited a direct co-expressional link with NFκB in humans (score 0.046) and experimentally (score 0.062). Moreover, AKT-1 and TNF displayed putative homologous experimental interactions. TNF-α and NFκB interactions emphasized co-expressional links with a score of 0.0888 in humans and putative homologs in other organisms with a score of 0.063, as shown in Figure 7. One of the displayed direct co-expressional proteins for SDF1/CXCR4 interaction was the T-cell surface glycoprotein CD4. It also showed direct co-expressions with TNF-α with a score of 0.098, as shown in Figure 8. Moreover, Relb proto-oncogene, NFκB subunit (RELB), NFκB inhibitor alpha (NFKBIA), and tumor necrosis factor receptor superfamily member 1A (TNFRF1A) showed direct co-expressional interactions with TNF-α and NFκB, as shown in Figure 8. 

## 3. Discussion

Cisplatin is a commonly used chemotherapeutic regimen for solid tumors. However, given its association with serious adverse effects such as nephrotoxicity (especially in higher doses), long-term carcinogenicity, and the development of acquired resistance to its apoptotic effects, as well as its lack of antiangiogenic therapeutic properties, caution is needed in its use [33,34,35].

The new molecular chemotherapeutic discoveries for current known plant extracts are important economic and pharmacovigilant trends. As a pentacyclic triterpene saponin found in Hedera or Nigella species, α-hederin is a promising active ingredient [36]. It provided various chemotherapeutic potentials in different in vitro and in vivo cancer models, including gastric cancer cells [26,37], colon cancer cells [38], breast cancer cells [24], and the human ovarian cancer cell line SKOV-3 [36]**,** and it was also proven to provide anti-oxidant activity [39]. Few studies have tested the chemotherapeutic potential of α-hederin: research tends to focus on biological activity rather than mechanisms.

In the current study, we used the EST experimental model, which is widely used for testing the chemotherapeutic potential of drugs [40,41,42]. The molecular individual and combined therapeutic roles of α-hederin with cisplatin were probed in the current study. We found that α-hederin was able to enhance the chemotherapeutic potential of cisplatin, which appeared as a reduction in the mass of the solid tumors. This action occurs, at least partly, via the downregulation of SDF1/CXCR4/*p*-AKT/NFκB-manipulated inflammatory, proliferative, and invasiveness signals. In addition, α-hederin alters the physical occurrence of EST.

In fact, the chemokine–chemokine receptor interactions of SDF1 and CXCR4 has been emphasized [43]. Different signaling paths with translational impacts in cancer-stem-like cells have been recognized in SDF1/CXCR4 interactions, including AKT-1 [44]. We found this ordered signaling path in the current work as well. Moreover, we found direct ordered interconnections with TNF-α and/or NFκB via both bioinformatic approaches that used verified databases and our in vivo mouse model of EST. Previous studies demonstrated the interconnection of SDF1/CXCR4 and TNF-α in cancer angiogenesis and metastasis [45,46]. Furthermore, AKT-1 has been found to influence, in a co-expressional manner, the translation and activation of NFκB [47], which we corroborated in the present study. TNF-α and TNFRF1A demonstrated a translational impact on NFκB in the current computational analysis, which agrees with previously published knowledge [48,49].

In mice inoculated with ESTs, the masses of the tumors declined after cisplatin and/or α-hederin administration. These data reflect the antitumor potential of α-hederin compared to cisplatin. Similarly, in SW620 cancer colon cells, the reduction in viable cells and pausing of the cell cycle G2 and M phases were confirmed after α-hederin exposure [38]. In addition, apoptosis, which is dependent on mitochondrial activation and caspases and is regulated by cyclin D1, was enhanced by α-hederin [24,26,38,50]. Furthermoroe, α-hederin could manipulate cancer cell proliferation through phosphatidylinositol 3-kinase/AKT/c-Jun N terminal kinase signals [50]. Notably, synergistic apoptotic and antiproliferative chemotherapeutic responses were confirmed after supplementation of α-hederin and cisplatin in gastric cancer cells both in vitro and in vivo [37]. 

Many tumors are influenced by chemokine–receptor interactions in autocrine and paracrine patterns [8]. CXCR4 and its ligand SDF1 have previously been identified as important metastatic contributors in a variety of malignancies [51,52]. CXCR4 overexpression is linked to enhanced vascular endothelial growth factor recruitment, cancer cell proliferation, and metastasis [53,54]. Moreover, CXCR4 suppression has also been associated with increased tumor cell death [54,55]. 

To address current knowledge gaps, it will be necessary to investigate the SDF1/CXCR4 signaling pathway in ESTs. In the current work, EST tissues exhibited high expressional levels of SDF1/CXCR4. These insights will aid in understanding the integration of chemokines and their receptor distribution and affinities in tumor cell responses to therapy, as well as the mechanisms of invasiveness. Cisplatin has been found to inhibit the SDF1/CXCR4 axis and its impact on ovarian cancer metastasis [56]; these findings are consistent with our research findings. Moreover, in our research, α-hederin showed marked reduction in the expressional levels of SDF1/CXCR4 in the untreated EST tissues and with cisplatin. It should be noted that α-hederin might be taken concurrently to minimize chemotherapeutic resistance and improve therapeutic efficacy. 

In agreement with our work, previous research has shown that AKT inhibition has preventive and therapeutic potential with good prognosis in several cancers, including the ESTs. Moreover, AKT is partially modulated by SDF1/CXCR4 interactions [10,13,21,23], and α-hederin has shown reductive capabilities for oral cancer SCC-25 cell lines [57]. In the current work, although cisplatin was shown to reduce tumor *p*-AKT-1 expression, combining it with α-hederin improved this impact. 

In the cancer microenvironment, several proinflammatory and proangiogenic molecular signals are intercalated, where NFκB is a potential rapid transcriptional regulator. Upon stimulation by chemical, inflammatory, infectious, or carcinogenic cellular stresses, intranuclear translocation of NFκB emerges, ending in control of cellular survival and proliferation. It also adjusts different regulatory genes involving inflammatory, apoptotic, angiogenic, metastatic, and chemo- and radio-resistant genes [58,59,60]. Notably, SDF1/CXCR4 showed stimulatory impacts on NFκB activation and translocation [12]. Our current data demonstrated higher expressional levels of NFκB in EST tissues, which agrees with prior research [61,62,63]. Moreover, the NFκB-dependent transcriptional pro-/inflammatory pathways are partially dependent on cancer-associated fibroblasts and matrix metalloproteinases, which are responsible for the sustained inflammatory and oncogenic milieu [60,62,64,65]. 

In the present study, individual administration of α-hederin to EST-bearing mice resulted in marked reduction in the elevated expressional tumor levels of NFκB. Furthermore, concurrent exposure to cisplatin and α-hederin markedly reduced the tumor NFκB levels. Similarly, α-hederin prohibited the NFκB signals in the SW620 cancer colon cells. It blocked NFκB translocation to the nucleus through amelioration of the regulatory proteins [38].

Cisplatin chemotherapeutic resistance is a challenging problem in cancer patients. Some of the various resistance mechanisms include augmented cellular efflux pumps, DNA repairing, detoxification, and antiapoptotic signaling [66,67]. Across different EST studies, the addition of other herbal medications to cisplatin increased the therapeutic efficacy of individual cisplatin usage and decreased its resistance specifically by enhancing apoptotic signals [68,69,70,71]. In cisplatin-treated cancer cells, translocational and nuclear activation of NFκB were emphasized [72]. Presently, the administration of α-hederin countered the cisplatin-induced NFκB spur. This may provide a clue about how α-hederin could synergistically enhance the chemotherapeutic effects of cisplatin at the cellular levels. 

In the current EST experimental model, to show the synergistic effect of α-hederin with cisplatin, we assessed the SDF1, CXCR4, *p*-AKT, and NFκB molecular therapeutic pathways. The related proinflammatory and pro-apototic cytokines displayed significant reductions after individual and combined regimens of α-hederin with cisplatin. 

TNF-α is an important immunological and inflammatory cytokine. In EST-bearing mice, tumor TNF-α revealed marked increments. This pathological evidence could be elucidated by recruiting tumor-infiltrating macrophages and elevating the reactive oxygen species that result from the oxidative stress ecosystem [62,63,73,74,75]. Of notice in cancer immunogenesis, NFκB has crucial immune responses for both innate and adaptive types. Moreover, progressive inflammation and genetic instability found in cancer paracrine environments induced by immune-cell-generated reactive oxygen species have an activating role on NFκB [76,77,78,79]. These evidence blocks could illustrate the chemotherapeutic effects of α-hederin through the reciprocal relationship of elevated TNF-α and activated NFκB.

Moreover, a number of studies investigated α-hederin for its promising chemotherapeutic potential, since many research papers documented its cytotoxic action against cancer cell lines, including lung carcinoma, colon adenocarcinoma, larynx epidermoid carcinoma, and pancreas carcinoma [80,81,82,83,84], as well as in vivo tumors [85,86,87]. The cytotoxic action of α-hederin was thought to be mediated via promotion of apoptosis and alteration of membranes [88,89]. One study reported that α-hederin inhibits growth and induces apoptosis in breast cancer cells [90], while another mentioned that α-hederin induces autophagy and cell death in colorectal cancer cells via an ROS-dependent mechanism in breast cancer cells [24] and AMPK/mTOR signaling pathway activation [25]. In the current study, we evaluated the activity of α-hederin on breast solid tumor growth and apoptosis of ESTs grown in mice and explored the underlying mechanisms.

## 4. Materials and Methods

### 4.1. Computational Interaction Analysis for the Target Proteins

To investigate the targeted protein–protein interaction, the STRING database (https://string-db.org (accessed on 21 February 2023), Version 11.5) was used on 27 September 2022. Through our search, the targeted proteins and the chemokine–chemokine receptor pathways were scoped, including SDF1 (CXCL 12), CXCR4, AKT-1, NFκB, and TNF-α. The search was limited to “Homo sapiens”. The interaction score was restricted to 0.700, a high confidence score. The evidence interaction types between the nodal proteins were represented by the network edges, where these interactions were experimentally proved or not, and the gene interaction predictability. The edge interactions were visualized with colors: green for gene neighborhood interactions, red for gene fusion interactions, blue for gene co-occurrence, black for gene co-expression, and olive green for database citational interactions. The first shell displayed only five query interactors. 

### 4.2. Signaling Pathway Enrichment Analysis

The target pathway was selected using the online KEGG pathway database (http://www.genome.jp/kegg, accessed on 21 October 2022).

### 4.3. Experimental Animals

Moustafa Rashed Company provided twenty-eight female Swiss albino mice for the trial, which were 20–25 g in weight. Mice were kept in plastic cages at 22 ± 3 °C with a normal light–dark cycle. Before the experiment began, mice were given a week to become used to their living arrangements. Food and water were always available during the course of the experiment. The experimental methods followed the ARRIVE guidelines and the National Research Council’s Guide for the Care and Use of Laboratory Animals. This research paper was approved by the Ethics Committee at Suez Canal University (202302RA3). 

### 4.4. Drugs and Reagents

α-Hederin (purity 98%; chemical formula: C41H66O12) was bought from Sigma-Aldrich, St. Louis, MO, USA) and dissolved in sunflower oil; oral administration was done in a volume of 0.1 mL/mouse. Cisplatin vials (Unistin vials, EIMC United Pharmaceuticals, Cairo, Egypt) were purchased from an oncology pharmacy and diluted to prepare a stock solution with sterile saline.

### 4.5. Tumor Cell Preparation and Intradermal Inoculation 

Ehrlich ascites carcinoma cell line was obtained from the National Cancer Institute (Cairo, Egypt). Ehrlich ascites carcinoma originated from a murine spontaneous breast cancer used to develop an ascites variant. The tumor cell line was preserved by intraperitoneal passage into another mouse after seven days [91]. Each mouse was inoculated subcutaneously at two sites on the lower ventral side (after shaving and disinfection with alcohol) with a 100-µL dose of Ehrlich carcinoma cell suspension (2.5 million cells per 0.1 mL) on each site [92,93].

### 4.6. Study Groups

The mice were split into four groups at random, with 10 animals in each. Group (1) was the EST control group. Group (2) mice were given an oral daily dose of α-hederin (80 mg/kg) [94]. Group (3) mice were given cisplatin (4 mg/kg, intraperitoneally) twice weekly for 21 days. Group (4) mice were given a combination of cisplatin and α-hederin at the same time as the monotherapy groups. All treatments were started at day 8 after tumor inoculation and continued for twenty-one days [95,96].

### 4.7. Harvesting the Solid Tumors

The mice were sedated with thiopental sodium (50 mg/kg) and killed by cervical dislocation at the end of the experiment. Tumor discs were dissected and weighed from both sides. The right disc was used to explore the histopathological and the immunohistochemical analyses. The left disc, on the other hand, was promptly frozen and stored at −80 °C till the time of biochemical experiments.

### 4.8. Western Blotting for p-Akt, SDF1, and CXCR4 Proteins 

The frozen tumor discs were disintegrated in RIPA buffer that contains protease and phosphatase inhibitors. To eliminate solid impurities, cell lysates were spun at 2000× *g* (20 min, 4 °C). The protein concentration in supernatants was assessed by using the Bio-Rad Quick Start^TM^ Bradford Protein Assay kit. After initial denaturation with 4× Laemmli Sample Buffer, same quantities of protein from EST’s homogenate were loaded on sodium dodecyl sulphate polyacrylamide gel (Bio-Rad, Hercules, CA, USA). Following electrophoresis, the transportation to the nitrocellulose membranes was held to the separated gel’s protein. To block the free sites on the membranes, they were incubated for 1 h in 5 percent nonfat dried milk (Bio-Rad). Then, incubation with primary antibodies for targeted proteins at a dilution of 1:500 and 4 °C was conducted: rabbit polyclonal antibodies (SDF1 antibody [N1C3] Gene Tex, Irvine, CA, USA, Cat No. GTX116092), rabbit CXCR4 polyclonal antibodies (Proteintech Group Inc., Rosemont, IL 60018, USA, Cat Number: 11073-2-AP), and p-AKT polyclonal antibody (catalog #sc-293125, Santa Cruz Biotechnology Inc., Dallas, TX, USA). After washing, the blots were treated with horseradish peroxidase (HRP)-conjugated secondary antibody and goat anti-mouse. The protein was detected using the ECL substrate Western blotting detection kit (#170-5060, Biorad) with enhanced chemiluminescence. Then, we captured the film bands by a charge-coupled device with a camera-based imager (ImageQuantTMLAS500, GE Healthcare Life Sciences, Marlborough, MA, USA). ImageJ software (NIH) was used to determine the intensity of immunoreactivity [97,98]. 

### 4.9. RT-PCR Quantification of Cytokines and Factors in ESTs

Tumoral total RNA was extracted by the aid of RNArasy Mini Kit from Qiagen Company (Hilden, Germany). We assessed RNA concentrations and purity using a NanoDrop ND-1000 spectrophotometer (NanoDrop Tech., Inc. Wilmington, DE, USA). We then transformed the total RNA into cDNA using a high-capacity cDNA reverse transcriptase kit (Applied Biosystems, Waltham, MA, USA). Then, we performed the PCR in a 48-well plate from a Sybr Green I PCR Master Kit (Fermentas, MA, USA) using the Step One instrument (Applied Biosystems). Gene expression was calculated using the comparative Ct method [99]. Table 1 shows the primer sequence for the measured genes.

### 4.10. ELISA Quantification of Cytokines in EST Homogenates 

Samples from the frozen tumor discs were analyzed by enzyme linked immunoassay (ELISA) to quantify the tumors’ TNF-α (Mouse TNF- α ELISA Kit, Cat. Number: MBS2500421, MyBiosource, San Diego, CA, USA) and NFκB (Mouse NFκB RTU ELISA Kit, Cat number: MBS4501353, MyBiosource). ELISA reader (tat Fax 2100, USA) was employed to measure the color intensity at the specified wavelength. 

### 4.11. Tumor Histopathology and Assessment

The harvested right tumor discs were preserved in a 10% phosphate-buffered formaldehyde solution (Al-nasr Company, Helwan, Egypt). After that, the formalin-fixed tissues were encased in paraffin wax and sectioned at 4 micrometer intervals (Leica microtome RM 2135, Leica Instruments GmbH, Nussloch, Germany). After that, the slices were stained with hematoxylin and eosin (H-E) and inspected blindly. Photographs were acquired at 100× and 400× original magnification (objective 10×, 40×), using the UIS optical system (Olympus^®^, Shinjuku, Japan). Tumors were assessed for the count of giant cells and mitotic pictures in addition to area of necrosis % [40,93]. 

### 4.12. Immunohistochemical Imaging and Quantification of NFκB in Tumor Tissue 

Tumor sections were deparaffinized and then incubated with 3% H_2_O_2_ to block the activity of endogenous peroxidases. Then, antigen retrieval was conducted by covering the tissues by citrate buffer and boiling. Primary rabbit polyclonal antibodies against mouse NFκB (1:100, Thermo Scientific, Waltham, MA, USA) were used and applied to the tumor tissues overnight. After that, we washed the tumor sections 3 times and used the Power-Stain^TM^ 1.0 Poly horseradish peroxidase (HRP)-DAB kit (Genemed Biotechnologies, San Francisco, CA, USA). Finally, all slides were cover-slipped and then examined blindly by a pathologist, and images were taken and analyzed using ImageJ software (Bethesda, MA, USA). 

### 4.13. Data Handling and Statistical Analysis

The statistical package for social sciences (SPSS) application was used to process the statistical data (windows version number 25). Data were reported as mean ± SD. We used one-way analysis of variance (ANOVA) and the Bonferroni test for pair-wise comparison. We set the accepted level of significance as *p* < 0.05.

## 5. Conclusions

This study documented the chemotherapeutic potential of α-hederin when used as a monotherapy and revealed that α-hederin augmented the potential of cisplatin against ESTs grown in mice. α-hederin suppressed the SDF1/CXCR4/*p*-AKT/NFκB signaling that was, at least partly, responsible for mitigating the growth of ESTs. Further studies are recommended to investigate the chemotherapeutic potential of α-hederin in other models of cancer in rodents and to test the possible alleviation of cisplatin-induced organ toxicities. 

## Figures and Tables

**Table 1:** Primer sequences of genes measured in the study.

Gene	Forward	Reverse
TNF-α	AGAACTCCAGGCGGTGTCTGT	CCTTGTCCCTTGAAGAGAACC
NFκB	AATTGCCCCGGCAT	ATGCGCCAATGCCCT
Caspase 3	ATGTCAGCTCGCAATGG	AAGAAATTATGGAATTG
GAPDH	TGGCACAGTCAAGGCTGAGA	CTTCTGAGTGGCAGTGATGG

## Data Availability

Data are available from the authors upon request.
